# Editorial: Human-robot interaction for children with special needs

**DOI:** 10.3389/frobt.2023.1206079

**Published:** 2023-09-13

**Authors:** Alireza Taheri, Adham Atyabi, Ali Meghdari, Minoo Alemi

**Affiliations:** ^1^ Social and Cognitive Robotics Laboratory, Center of Excellence in Design, Robotics and Automation (CEDRA), Sharif University of Technology, Tehran, Iran; ^2^ NeuroCognition Laboratory, Department of Computer Science, University of Colorado Colorado Springs, Colorado Springs, CO, United States; ^3^ Department of Humanities, West Tehran Branch, Islamic Azad University, Tehran, Iran; ^4^ Chancellor Fereshtegaan International Branch, Islamic Azad University, Tehran, Iran

**Keywords:** social robotics, human-robot interaction (HRI), children with special care needs, children with special educational needs and disabilities, autism spectrum disorder, neurodevelopmental disorders, robot assisted activity/therapy

Utilizing robots in different areas such as education and cognitive rehabilitation of children with special needs (e.g., children with autism, cancer, dyslexia, hearing disabilities, *etc.*), is rapidly expanding worldwide (Esfandbod et al., 2022; Taheri, 2023). Hence, the use of this technology as a modern tool in diverse educational/treatment classes will be unavoidable in the next few years. Many studies have indicated the positive aspects of Human-Robot Interaction (HRI) platforms for children. For example, specially designed social robots with cute characteristics can facilitate effective engagement levels and compliance of children with special needs toward teachers/therapists and robots in robot-assisted classes (Ghorbandaei-Pour et al., 2018; Taheri et al., 2018; Taheri et al., 2021). Moreover, socially assistive robots could play a catalytic role in making the education and social/cognitive rehabilitation process of children more appealing in a friendly environment, and this could increase the attention span of the participants and their tendency to become deeply involved in educational/treatment tasks (Basiri et al., 2021a; Basiri et al., 2021b). Alternatively, educational and therapeutic robots could benefit teachers and clinical supervisors in several ways, such as by supporting real-time treatment assessment and keeping records of the children’s performance in robot-assisted sessions (Hosseini et al., 2021; Esfandbod et al., 2023; Taheri et al., 2023). Although there are many studies that shed light on using robots for educating/rehabilitating children with special needs, there are still many challenges in this field to be addressed. This Research Topic addressing specific different challenges as well as positive/negative aspects of HRI for children with special needs makes up six of the latest studies in Frontiers in Robotics and AI.

## Published papers in this Research Topic

Of the six research papers presented on this Research Topic, three have been conducted with children with autism. Chevalier et al. investigated the effect of sensory sensitivity during robot training in children with ASD. The authors observed that visual and auditory sensitivity influenced improvements in the ability to initiate joint attention. Vora et al. investigated the affordability of developmentally appropriate toys and play areas provided by mobile SARs. The purpose of this study was to assess the role of SARs on children’s physical activity, play behavior, and toy use behavior during free play. The results of this research suggest that a mobile SAR offers affordability through rewards that elicit more time for children’s interaction with the SAR and free play. Baraka et al., suitable action sequences in robot-assisted autism therapy have been investigated. The authors found that an exploratory study of eleven children with autism highlighted the usefulness and limitations of different modes in relation to a variety of possible interaction goals. They demonstrated that personalized robots could be used in the short- and long-term. They mentioned that it paved the way to balance the objective goals in such assistive therapies. Zehfroosh and Tanner, the authors presented a novel hybrid probabilistic approximation reinforcement learning algorithm for Markov decision processes that intelligently retains the advantageous features of both model-based and model-free methods. An experimental implementation of DDQ in the context of pediatric motor rehabilitation facilitated by interaction between infants and robots highlighted the potential benefits of the described method. Ligthart et al. described two studies of children learning about autonomous SARs. The authors found similarities between how children form relationships with humans and how children form relationships with robots. And Feng et al., a robot-assisted platform for music therapy of children with ASD was developed and investigated.
